# Zimbabwe's national AIDS levy: A case study

**DOI:** 10.1080/17290376.2015.1123646

**Published:** 2016-01-18

**Authors:** Nisha Bhat, Peter H. Kilmarx, Freeman Dube, Albert Manenji, Medelina Dube, Tapuwa Magure

**Affiliations:** ^a^ is an International Experience and Technical Assistance Fellow affiliated to US Centers for Disease Control and Prevention, Harare.; ^b^ MD, is a medical epidemiologist affiliated to US Centers for Disease Control and Prevention, Harare.; ^c^ is affiliated to Division of Global HIV/AIDS, US Centers for Disease Control and Prevention, Atlanta; ^d^ is a Research and Documentation Coordinator affiliated to National AIDS Council of Zimbabwe, Harare; ^e^ is a Finance Director affiliated to National AIDS Council of Zimbabwe, Harare; ^f^ is a Communications Director affiliated to National AIDS Council of Zimbabwe, Harare; ^g^ MBcHB, MPH, MBA, is a Chief Executive Officer affiliated to National AIDS Council of Zimbabwe, Harare

**Keywords:** health care financing, HIV, AIDS, Zimbabwe, financement des soins de santé, le VIH, le sida,, le Zimbabwe

## Abstract

*Background*: We conducted a case study of the Zimbabwe National AIDS Trust Fund (‘AIDS Levy’) as an approach to domestic government financing of the response to HIV and AIDS.

*Methods*: Data came from three sources: a literature review, including a search for grey literature, review of government documents from the Zimbabwe National AIDS Council (NAC), and key informant interviews with representatives of the Zimbabwean government, civil society and international organizations.

*Findings*: The literature search yielded 139 sources, and 20 key informants were interviewed. Established by legislation in 1999, the AIDS Levy entails a 3% income tax for individuals and 3% tax on profits of employers and trusts (which excluded the mining industry until 2015). It is managed by the parastatal NAC through a decentralized structure of AIDS Action Committees. Revenues increased from inception to 2006 through 2008, a period of economic instability and hyperinflation. Following dollarization in 2009, annual revenues continued to increase, reaching US$38.6 million in 2014. By policy, at least 50% of funds are used for purchase of antiretroviral medications. Other spending includes administration and capital costs, HIV prevention, and monitoring and evaluation. Several financial controls and auditing systems are in place. Key informants perceived the AIDS Levy as a ‘homegrown’ solution that provided country ownership and reduced dependence on donor funding, but called for further increased transparency, accountability, and reduced administrative costs, as well as recommended changes to increase revenue.

*Conclusions*: The Zimbabwe AIDS Levy has generated substantial resources, recently over US$35 million per year, and signals an important commitment by Zimbabweans, which may have helped attract other donor resources. Many key informants considered the Zimbabwe AIDS Levy to be a best practice for other countries to follow.

## Résumé

*Contexte*: Nous avons effectué une étude de cas sur le Fonds National du SIDA au Zimbabwe («Le Levy SIDA») pour illustrer une approche de financement en fidéicommis par un gouvernement national, en réponse à l’épidémie de VIH/SIDA.

*Méthodes*: Les données proviennent de trois sources : une revue de la littérature, incluant une recherche de la littérature grise, l'examen de documents gouvernementaux émanant du Conseil National du SIDA au Zimbabwe, et des entretiens avec des informateurs clés représentant le gouvernement du Zimbabwe, la société civile et les organisations internationales.

*Résultats*: La recherche documentaire a retourné 139 sources, et 20 personnes ont été interrogées. Créé par la législation de 1999, le fonds Levy SIDA est financé par un impôt sur le revenu de 3% pour les particuliers et une taxe de 3% sur les profits des employeurs et des fonds d'investissement (qui excluaient l'industrie minière jusqu'en 2015). Il est géré par le Conseil National du SIDA, un organisme parapublic, grâce à une structure décentralisée impliquant les comités d'action contre le sida. Les revenus ont augmenté depuis la création de 2006 à 2008, une période d'instabilité économique et d'hyperinflation. Suite à la dollarisation en 2009, les revenus annuels ont continué d'augmenter, atteignant 38.6 millions de US$ en 2014. Par décret, plus de 50% des fonds sont utilisés pour l'achat de médicaments antirétroviraux. Les autres dépenses comprennent les coûts d'administration et d'investissement, la prévention du VIH, le suivi et l'évaluation. Plusieurs systèmes de contrôles financiers et d'audit sont en place. Les informateurs clés ont perçu la taxe sida comme une solution «maison» qui a permis l'appropriation nationale et a réduit la dépendance envers des bailleurs de fonds. Cependant, ces informateurs ont appelé à une transparence accrue, à la responsabilité financière, et à une réduction des coûts administratifs du fonds Levy SIDA. Ils ont égalent recommandé des changements visant à augmenter les revenus.

*Conclusions*: Le fonds Levy SIDA du Zimbabwe a généré d'importantes ressources, soit plus de 35 millions de US$ par an sur la période récente, tout en signalant un engagement important des Zimbabwéens, ce qui peut avoir contribué à attirer d'autres donateurs. De nombreux informateurs clés considèrent le fonds Levy SIDA du Zimbabwe comme un exemple de bonne pratique qui devrait être suivi par d'autres pays.

## Introduction

Financing the response to HIV infection and AIDS is particularly challenging in resource-poor countries that have a high burden of disease (Ávila *et al*. 2013; Izazola-Licea *et al*. 2009; UNAIDS and Kaiser Family Foundation 2014) Funding sources include international assistance from bilateral donor governments, multilateral institutions, the private sector, charities, foundations, religious organizations, and households and individuals (UNAIDS and Kaiser Family Foundation 2014). In 2006, 87% of the HIV and AIDS response funding for 17 low-income countries came from international donors (Izazola-Licea *et al*. 2009). Low- and middle-income country governments have played an increasing role, although their response has varied among countries and over time. Low- and middle-income countries spending from government sources increased three-fold from US$2.1 billion in 2000 to US$6.6 billion in 2010, with public funding levels positively associated with per capita income and HIV infection prevalence (Ávila *et al*. 2013).

Zimbabwe, a low-income, land-locked country in southern Africa with a population of 13 million, has been hard hit by HIV infection with a peak prevalence of 26% in adults in the late 1990s (Global AIDS Response Country Progress Report, Zimbabwe 2014). In 1999, the Zimbabwean government introduced a National AIDS Trust Fund or ‘AIDS Levy' to fund HIV and AIDS prevention, care and treatment in response to a growing rate of HIV infection and AIDS and limited government funding to combat the problem.

We conducted a case study of the Zimbabwe AIDS Levy in order to describe: (1) the legislative history, (2) the roles of government agencies and external partners, (3) historical budget figures, (4) how and what the AIDS Levy finances, (5) financial controls, and (6) perceived strengths, weaknesses and potential areas for improvement. Findings are intended to strengthen the Zimbabwe AIDS Levy and also have wider application outside of Zimbabwe where other countries can consider adopting similar polices to finance their response to HIV and AIDS.

## Methods

The paper is a qualitative case study of a public policy and health financing mechanism (Hanney *et al*. 2003). The case study utilized three forms of data collection: (1) a literature review to identify existing literature on Zimbabwe's AIDS Levy; (2) document review of financial documents, strategic plans, and monitoring and evaluation reports provided by the Zimbabwe National AIDS Council (NAC); and (3) key informant interviews with representatives of the Zimbabwean government, civil society and international organizations familiar with the AIDS Levy.

First, we reviewed published peer-reviewed and gray literature on the AIDS Levy and the NAC. We searched Pub-Med and Google Scholar using the terms ‘Zimbabwe AIDS levy' and ‘Zimbabwe National AIDS Council'. Gray literature documents on the AIDS Levy were provided by NAC and key informants and included reports done by organizations including NAC, Southern African Development Community (SADC) and universities. Second, a review was conducted of government documents provided by NAC collaborators. The types of documents reviewed included national policies and guidelines, financial documents, budgets, audit reports, monitoring and evaluation documents, and documents on programmatic planning.

Lastly, key informant interviews were conducted with three groups of stakeholders: representatives of Zimbabwean government, representatives of Zimbabwean civil society and representatives of international organizations. Key informants were selected purposively for their specialized knowledge or perspective on the AIDS Levy and represented a spectrum of viewpoints. Subsequent key informants were interviewed based on recommendations of initial key informants during their interviews. Key informant interviews took place from November 2012 to January 2013 in Zimbabwe.

Each interview participant was asked the same set of open-ended questions. Interviews were conducted in-person or by telephone, when an in-person interview was not feasible. The interviews were documented by note taking. Each interview broadly covered the following topics: the respondent's professional background and how it relates to the AIDS Levy; their knowledge of the AIDS Levy; their opinion on the strengths and weaknesses of the AIDS Levy; their recommendations to improve the AIDS Levy and whether they believed the AIDS Levy could serve as a model for other countries to adopt. Responses to the interviews were summarized with the participant responses to each question, anecdotes and interviewer's impressions, organized by question. Descriptive coding was the primary method of analysis of the key informant interviews. After four interviews, initial descriptive codes were developed on key themes, concepts, questions, and ideas. These were refined based on results of subsequent interviews and responses were synthesized to describe the role of different agencies in the AIDS Levy and perceived strengths and weaknesses of the AIDS Levy.

The study protocol was approved by the Office of Associate Director for Science of CDC Center for Global Health. Key informants provided verbal informed consent and were assured that neither their decision to participate or not, nor their responses would affect their employment status. Key informant responses are presented anonymously. Respondents did not receive any compensation for their participation.

## Findings

The literature search yielded 139 articles, chapters and reports that mentioned the Zimbabwe AIDS Levy and 39 that mentioned the Zimbabwe NAC. Gray literature included reports on the AIDS Levy done by organizations including NAC, SADC and universities. Relevant and informative publications and reports were used for this case study report (Baird 2006; Financial Statements of National AIDS Council For Year Ends 2003 to 2012; Gandure 2009; Garbus and Khumalo-Sakutukwa 2003; Government of Zimbabwe 1999a, 1999b, 1999c; Matchaba-Hove 2006; Mpofu and Nyahoda 2008; Price-Smith and Daly 2009; Rembe 2006; UNAIDS 2012). Interviews were conducted with a total of 20 key informants representing 13 different institutions. Seven interviewees represented the government of Zimbabwe; seven were from international organizations, four from civil society organizations, and two from organizations directly supported by the AIDS Levy.

### Legislative history

The idea of the AIDS Levy was developed in the early 1990s, but did not become law until 1999. A convergence of several political factors allowed for the successful enactment of the AIDS Levy. The enactment of the AIDS Levy reflected political will, engagement of stakeholders and collaboration between the Office of the President, Parliament, the Ministry of Finance, the Ministry of Health and Child Welfare (now called the Ministry of Health and Child Care [MOHCC]), and labor groups. Much of the political will and support for the AIDS Levy was garnered as it was viewed as a homegrown solution that could attract additional donor support. The AIDS Levy was modeled after a similar tax called the Drought Levy which had been enacted to support food imports during the 1992 drought, which allowed the public to already have familiarity with the mechanisms of such a levy.

Two key pieces of legislation established the policy framework of the AIDS Levy. In 1999, Parliament enacted Section 14, Subsection 14 and 15 of the Finance Act, Chapter 23:04, which established the National AIDS Trust Fund as a 3% income tax for individuals and 3% tax on profits of employers and trusts. The mining industry, though not its employees, was initially exempted from contributing to the AIDS Levy. Second, Parliament enacted the NAC of Zimbabwe Act in 2000, which established the responsibilities of the NAC, including the administration of the AIDS Levy. By law, NAC was established as a non-profit, parastatal organization. The NAC Act established the NAC Board, a multi-sectoral Board, appointed by the President that develops the annual strategic plan for NAC. The AIDS Levy and NAC legislation along with the National HIV and AIDS Policy and National HIV and AIDS Strategic Framework in 1999 were the key foundations for AIDS policy in Zimbabwe. The 2015 National Budget calls for extension of the AIDS Levy to the mining industry (Ministry of Finance and Economic Development 2014).

### Agencies’ roles

There are several governmental and parastatal agencies involved in the collection, management and distribution of the AIDS Levy. The NAC Board is 14-member, multi-sectoral body, appointed by the President, in consultation with the MOHCC, The NAC Board has a diverse membership representing a spectrum of societal perspectives, including the Permanent Secretary of the MOHCC, CEO of NAC, representatives from Traditional Medical Practitioners’ Council, the Law Society of Zimbabwe, health care providers, women, youth, religious groups, and representatives of groups of people living with HIV, commerce and trade unions. The NAC Board is responsible for approving general operational policies and AIDS Levy Budget consistent with the strategic framework annually.

The NAC staff administers the AIDS Levy and manages the daily operations of the AIDS Levy. The responsibilities of NAC staff include coordinating, providing support and monitoring the decentralized, multi-sectoral response to HIV and AIDS in accordance with the National HIV and AIDS Strategic Framework and the annual strategic framework established by the NAC Board. In addition, NAC staff promote monitoring and evaluation and research, and procure commodities and equipment including antiretroviral medication (ARV) and laboratory equipment.

A unique aspect of the AIDS Levy is that many of the activities supported are conducted through a decentralized structure of AIDS action committees that administer some AIDS Levy funds. Committees exist at provincial and district levels. In 2015, each of the 10 provinces is receiving US$500,000 to support implementation of HIV prevention interventions by the districts. Ward focal persons collect data and coordinate activities at the community level.

The Zimbabwe Revenue Authority (ZIMRA) collects and transfers the AIDS Levy directly to the National AIDS Trust Fund on a monthly basis. Unlike other taxes that are managed by the Ministry of Finance, the AIDS Levy funds are directly transferred to the National AIDS Trust Fund to be used only for the purpose of the HIV and AIDS response. The MOHCC is involved in several aspects of the AIDS Levy; it approves its annual work plan and budget for the AIDS Levy, implements programs funded by the AIDS Levy, and participates in monitoring and evaluation activities under NAC coordination. The Ministry of Public Service, Labor and Social Welfare implemented one of the programs supported by the AIDS Levy, Basic Education Assistance Module that provided support to orphans and vulnerable children.

### Revenues

The revenues and success of the AIDS Levy has depended on the strength of the economy. The AIDS Levy grew steadily from its inception in 2000 until 2006 through 2008, when Zimbabwe was faced with significant economic instability and hyperinflation. During the period of economic instability the AIDS Levy continued, though its purchasing power was limited and due to extreme hyperinflation, and was ultimately rendered essentially valueless. In February 2009, the Zimbabwean government abandoned the Zimbabwean dollar and switched to the US dollar. As the economy stabilized, the AIDS Levy began to grow and collected US$38.6 million in 2014. The annual AIDS Levy collection amounts are listed in Table 1. Based on estimates of the AIDS Levy collection amounts provided by the Ministry of Finance, the NAC Board determines the annual budget and how the AIDS Levy funding will be allocated toward the HIV and AIDS response.

**Table 1.** Annual revenue of the Zimbabwe AIDS Levy – 2000–2013.

Year	Annual revenue	Amount in USD^a^
2000	Zim$793,903	20,892
2001	Zim$1,525,922	27,744
2002	Zim$2,835,887	52,516
2003	Zim$11,247,338	13,868
2004	Zim$92,622,847	17,358
2005	Zim$262,537,228	27,124
2006	Zim$5,648,585,976	56,168
2007	Zim$1,118,870,698,065	
2008	Zim$347,482,953,678,240	
2009	US$5,710,820	
2010	US$20,522,121	
2011	US$26,459,054	
2012	US$32,640,678	
2013	US$34,236,005	
2014	US$38, 651,392	

^a^Zimbabwe dollar amounts converted to US dollars based on interbank exchange rates from June 30th of each year, 2000–2006.

### Funded activities

**Fig. 1.**
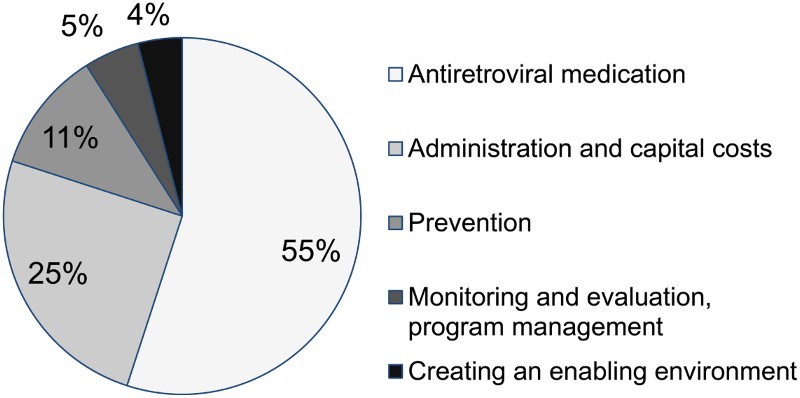
AIDS Levy budget allocations – 2009–2012.

Strategic activities in the following categories receive funding from the AIDS Levy based on the allocations shown in Fig. 1, which have been in place since 2009: ARV, prevention, monitoring and evaluation and program management, creating an enabling environment, and administration and capital costs.

Since 2005, the majority of the AIDS Levy has been directed toward the procurement and distribution of ARV. By policy, at least 50% of the AIDS Levy is allocated toward the purchase of ARV, with NAC is responsible for their procurement through guidance from the MOHCC on the ARV needs for the upcoming year, NAC follows the Zimbabwe's procurement laws under the State Procurement Act to purchase the appropriate drug supply. NAC follows a formal tender process for larger procurements (procurements greater than US$300,000) and competitive bidding with minimum of three bidders for procurements that are less than US$300,000. An adjudication process with the MOHCC, the Medicine Control Authority of Zimbabwe, and the National Pharmaceutical Company is conducted to award the procurement. The purchased drugs are then managed and distributed by the National Pharmaceutical Company together with donor-procured ARV.

Under the prevention programs, the AIDS Levy supports condom promotion, prevention of mother-to-child transmission of HIV, safe blood, youth and workplace programs, and more recently initiated support for voluntary medical male circumcision programs.

The other strategic areas that the AIDS Levy supports are programs to support creating an enabling environment, which involves support for advocacy for people living with HIV and AIDS and programs that support gender mainstreaming, orphans and vulnerable children, and the meaningful involvement of people living with HIV and AIDS. The AIDS Levy has directly supported advocacy groups including Zimbabwe National Network of People living with HIV and Southern Africa HIV and AIDS Information Dissemination Service.

The AIDS Levy supports monitoring and evaluation projects conducted by NAC in collaboration with the MOHCC, including the production of annual and quarterly monitoring and evaluation reports. Finally, the AIDS Levy supports administration of NAC, including capital costs, management and salaries.

### Financial controls

There are several financial controls in place to ensure the effectiveness of the AIDS Levy. ZIMRA and NAC validate that the amount collected by ZIMRA is the amount that NAC can account for in the National AIDS Trust Fund. Several audits of NAC's financial statements are conducted, including annual audits conducted by Zimbabwe's Office of the Comptroller and Auditor General and internal audits by NAC-employed auditors for all provinces and districts every two years. Furthermore, NAC provides quarterly financial reports to the MOHCC, the Ministry of Finance, and Parliament. The NAC Board Finance Committee reviews financial information quarterly. In addition, an audited annual report is publicly distributed and posted on the NAC website. External auditors such as the Local Fund Agent of the Global Fund have reviewed audits and financial reports as part of their capacity assessments.

In 2012, there was a series of protests and communications from Zimbabwean AIDS activists calling for increased accountability and transparency in the administration of AIDS Levy funds. In response, NAC re-published the most recent audited financial statements in the popular press. NAC representatives also held stakeholders meetings which included representatives of AIDS service organizations and of groups of people living with HIV to determine program gaps. One result of those meetings was the initiation of community monitoring quarterly assessments of antiretroviral treatment clinics. Another outcome was a series of quarterly meetings with news editors.

### Perceived strengths

From the key informant interviews, several themes were identified on perceived strengths of the AIDS Levy. One theme was the tangible benefits of the use of the funds, specifically that the majority of the funds were being used to purchase ARV. Secondly, the nature of the AIDS Levy as a ‘homegrown' solution which provided country ownership and reduced dependence on donor funding was seen as an important strength. Related to this, key informants reported that the creation of NAC and the AIDS Levy contributed substantially to the coordination of the national response by creating coordination structures at subnational levels that are responsible for planning, data collection, and reporting. Lastly, the basis of the AIDS Levy as an entity created by laws and not simply by administrative policy was also seen as a strength.

### Perceived weaknesses

Several themes were also identified from the key informant interviews regarding weaknesses of the AIDS Levy. The need for more transparency and accountability was a common concern, despite the regular reporting described above. One respondent said, ‘We know 50 percent of the money goes for ARVs, but we don't know what the other 50 percent goes towards.' Related to this, there was a perception that the administrative costs were too high and objections to provisions of the NAC Act, which, like other parastatal organizations in Zimbabwe, catered for benefits for employees such as loans for houses. Other reported weaknesses related to the tax burden of the AIDS Levy on working Zimbabweans and that the informal sector was not contributing. Lastly, there were related concerns about the availability of resources. Since the revenue of the AIDS Levy depended on the vibrancy of the economy, the resources to date have been insufficient. In the words of one respondent, ‘There is always not enough money. Not enough cake. There is constant competition for resources.'

### Key informants’ recommendations

The themes of the recommendations from key informants related closely to the perceived weaknesses. Key informants suggested that communication from NAC be improved to further promote accountability and transparency. A specific recommendation was to make available the quarterly reports that are submitted in Parliament. A second general recommendation was to reduce the administration and overhead costs funded through the AIDS Levy and increase the proportion of the funds dedicated to procurement of commodities and program implementation. The last thematic area of recommendations related to increasing the revenue for the AIDS Levy. Specific recommendations included increasing revenue from informal sector through a value added tax, from employers from which tax was only levied on profits, and from the mining sector (which was exempted as the time of the interviews).

Finally, key informants were asked if they thought the Zimbabwe AIDS Levy should be identified as a best practice which could be recommended for replication in other countries. Many responded positively; comments included: ‘In principle, it is a best practice.' ‘Yes, it removes a culture of dependency.' ‘It should be a model that should be replicated and studied so it can be enhanced.' One respondent endorsed specific features of the Zimbabwe AIDS Levy: ‘Countries should be encouraged to have a health fund. The fund should be administered by an independent board. They should have the courage to administer and audit.' Other key informants had a qualified response, for example: ‘It depends on the country. It could be taken out of the budget for health if the country has a well-managed budget.' ‘It depends on the economy of the country.'

## Discussion

This case study documents the establishment of the Zimbabwe AIDS Levy through legislation in 1999 and key elements of its subsequent implementation. Through a 3% tax on formal sector income and business profits (which excluded the mining sector until 2015), the AIDS Levy has raised well over US$100 million for the national response to HIV and AIDS, including US$38.6 million in 2014 alone. Multiple Zimbabwean government agencies are engaged in the administration of the AIDS Levy, led by the NAC. While half of the funds are used for procurement of ARV, other activities including prevention and monitoring and evaluation are implemented through a decentralized structure down to the village level. While key informants had suggestions to strengthen the AIDS Levy including increasing transparency and accountability and reducing administrative costs, the AIDS Levy was generally endorsed as a potential best practice for other countries to consider emulating.

How did the existence of the AIDS Levy impact the overall response to HIV and AIDS in Zimbabwe? While other low- and middle-income countries have committed increasing resources to the HIV and AIDS response, the Zimbabwe AIDS Levy is notable for having been established in the late 1990s, relatively early in comparison to the responses and funding from other low-income countries (Ávila *et al*. 2013). Zimbabwe is also notable for having a substantial decline in HIV incidence in the late 1990s and decline in adult HIV prevalence from 26% in the late 1990s to 15% in the early 2010s (Global AIDS Response Country Progress Report, Zimbabwe 2014). While the decline may not be attributed directly to the AIDS Levy, both are indicative of a vigorous national response to the epidemic. The AIDS Levy stands out as a well-established, highly visible commitment on the part of government as well as citizens. Most formal-sector employees have an itemized deduction for the AIDS Levy listed on each pay stub. As envisioned at inception of the AIDS Levy, substantial other donor support for the HIV and AIDS response has been successfully attracted to Zimbabwe, including currently each year over US$100 million from the Global Fund to Fight AIDS, Tuberculosis and Malaria and nearly US$100 million from the US President's Emergency Plan for AIDS Relief (UNAIDS and Kaiser Family Foundation 2014). Statements from the Global Fund indicate that the AIDS Levy was factor in the decision to increase funding for Zimbabwe (Zimbabwe Ministry of Health and Child Welfare 2013). The relative, direct public health impact of the AIDS Levy also needs to be considered together with other government spending. For example, US$38.6 million was raised by the AIDS Levy in 2014 while the overall MOHCC budget for 2014 was US$301 million, most of which funds the labor costs of health care workers who implement HIV and AIDS medical care and treatment. So, while the AIDS Levy is a very substantive commitment to the national response to HIV and AIDS, and it has funded some key non-health activities, it is a fraction of the overall national spending on health. Further policy considerations in this area should weigh the relative merits of the AIDS Levy versus the alternative of funding the response to HIV and AIDS from within the national budget derived from general revenue streams, taking into account the positive visibility of the AIDS Levy balanced against the administrative costs for a relatively small proportion of the overall response.

There are several methodological limitations of this case study. The first limitation is the lack of generalizability typically found in case study research. Secondly, much of the data collection and analysis were limited to the government documents made available. Some information may not be documented. Third, some key informants may have been biased or felt restricted in their responses due to perceived potential repercussions, which may have affected the study results. Finally, while a robust methodology was followed, the authors recognize that, due to the nature of qualitative research, alternative interpretations of the evidence are possible.

## Conclusion

The Zimbabwe AIDS Levy has been successful in raising substantial resources to address the epidemic of HIV and AIDS in Zimbabwe, recently over US$30 million per year. It is a visible indicator of a long-term commitment on the part of the government and citizens of Zimbabwe, and may have had a role in attracting other donor resources. Success factors have included the solid legal basis for the Levy, the engagement of multiple agencies as stakeholders, the decentralized implementation of some of the activities, and the financial controls over the use of the funds. Key informants recommended further increasing transparency, accountability and efficiency in the use of funds, and taking steps to increase the resources available. Many consider the Zimbabwe AIDS Levy to be a best practice which other countries may want to emulate.
